# Evolution of the SARS-CoV-2 spike protein in utilizing host transmembrane serine proteases

**DOI:** 10.1016/j.isci.2025.113318

**Published:** 2025-08-06

**Authors:** Aleksandra Milewska, Luis Fernando Cofas-Vargas, Adolfo B. Poma, Krzysztof Pyrć

**Affiliations:** 1 Virogenetics Laboratory of Virology, Malopolska Centre of Biotechnology, Jagiellonian University, Gronostajowa 7A, 30-387 Krakow, Poland; 2 Institute of Fundamental Technological Research, Polish Academy of Sciences, Pawińskiego 5B, 02-106 Warsaw, Poland

**Keywords:** Molecular interaction, Cell biology, Bioinformatics

## Abstract

SARS-CoV-2 entry into host cells depends on proteolytic activation of the spike protein by host proteases, a process shaped by spike mutations that influence viral specificity and infectivity. Using human airway epithelial models, this study investigated how different SARS-CoV-2 variants interact with host serine proteases. The Delta variant exhibited enhanced and stable binding to Hepsin through stronger ionic and hydrophobic interactions, promoting efficient spike activation and cell entry. In contrast, Omicron BA.1 showed weaker Hepsin binding and relied more on TMPRSS2 or cathepsins, depending on the cellular context. These findings reveal how variant-specific differences in protease usage are linked to spike protein mutations and cleavage site evolution. By illuminating the dynamic interplay between viral adaptation and host protease specificity, this work provides insights into mechanisms that influence viral transmission and immune evasion, with implications for developing targeted antiviral strategies and understanding the evolution of emerging SARS-CoV-2 variants.

## Highlights

• SARS-CoV-2 spike evolves to favor different proteases for activation over time
• Variant-specific entry relies on diverse TTSPs, not just TMPRSS2
• Spike mutations alter protease preference via sequence and structural changes

## Graphical abstract

**Figure:**
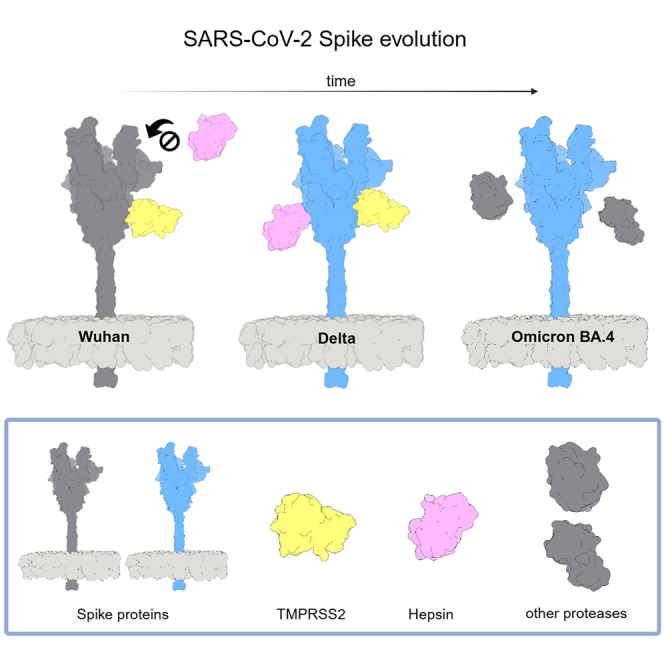

## Introduction

Severe acute respiratory syndrome coronavirus 2 (SARS-CoV-2) is associated with COVID-19, which caused a global pandemic in 2020. SARS-CoV-2 is an enveloped, positive-sense, single-stranded RNA virus belonging to the Betacoronavirus genus. SARS-CoV-2 is a close relative (79% genome sequence identity) to the SARS-CoV, which caused the 2002–2004 SARS epidemic.^1^ Other pathogens within this genus include human coronaviruses (HCoV) OC43 and HKU1, as well as the Middle East respiratory syndrome coronavirus (MERS-CoV).^2^^,^^3^^,^^4^^,^^5^^,^^6^ HCoV-NL63 or HCoV-229E, belong to the closely related alphacoronavirus genus.

The coronavirus virion comprises four structural proteins: nucleocapsid (N), membrane (M), envelope (E), and spike (S). Some betacoronaviruses also carry the hemagglutinin esterase on its surface, acting as the receptor-destroying enzyme. The spike protein is one of the most important, as it facilitates viral entry, mediating attachment to the host cell membrane and membrane fusion. At the molecular level, the spike protein is a trimeric, pyramid-shaped structure-oriented upside down and displayed on the viral membrane. The pre-fusion SARS-CoV-2 S protein consists of S1 and S2 subunits.^2^ Globular S1 facilitates receptor binding, while rod-like S2 is responsible for membrane fusion. The S1 subunit contains an N-terminal domain (NTD) and a receptor-binding domain (RBD) that primarily interacts with the angiotensin-converting enzyme 2 (ACE2) receptor, as well as with a recently identified receptor, TMEM106B, and neuropilin-1.^3^^,^^4^ Further, interactions have been reported between the SARS-CoV-2 S protein and cell surface glycans, which serve as attachment receptors.^3^^,^^4^^,^^5^ Receptor engagement by viral entry glycoproteins results in interactions with other cellular proteins, which induce significant conformational changes, bringing the membranes into proximity and triggering the fusion. Furin is a protein convertase broadly expressed in cellular protein synthesis and transport pathways. It cleaves the SARS-CoV-2 spike (S) protein at the S1/S2 site during viral protein biosynthesis and particle assembly, a process known as priming cleavage. While furin is the main enzyme responsible for this step, some S1/S2 cleavage still occurs in its absence, likely mediated by other related convertases.^6^^,^^7^

For SARS-CoV-2, for successful entry, fusion cleavage at the S2′ site is required. This process has been reported to be performed by the cell surface serine proteases and the endosomal cathepsins; however, the latter has been proven inefficient *in vivo*. TMPRSS2 (a type II transmembrane protein with serine protease activity) importance in respiratory virus infection, particularly for influenza viruses and SARS coronaviruses, is well established.^6^^,^^7^^,^^8^^,^^9^ TMPRSS2 is present in the gastrointestinal, respiratory, and urogenital epithelium and is believed to participate in processes like epithelial homeostasis and mucosal immunity.^10^ Also, it is androgen regulated, and the TMPRSS2-ERG gene fusion, resulting from a chromosomal rearrangement, is a hallmark of many prostate cancers, suggesting TMPRSS2 may contribute to tumor progression.^11^ Among these tissues, type II pneumocytes, ileal absorptive enterocytes, and nasal goblet secretory cells are major cell types that co-express TMPRSS2 and ACE2.^12^^,^^13^^,^^14^ Although TMPRSS2 is the most studied protease in coronavirus entry, other serine proteases in the lung, including human airway trypsin-like protease (HAT), TMPRSS4, TMPRSS11A, TMPRSS11E, matriptase, and secreted neutrophil elastase, also contribute to respiratory virus infections.^15^ Although SARS-CoV-2 is activated *in vivo* by TMPRSS2, the S2′ site can also be processed by cathepsins, particularly cathepsin L. In cell lines lacking the TMPRSS2 and expressing high levels of cathepsins, the ACE2-bound virus is internalized via clathrin-mediated endocytosis into the late endolysosome, where cathepsins cleave the S2′ site.^16^^,^^17^ The binding of SARS-CoV, SARS-CoV-2, or purified S protein to ACE2 induces ACE2 endocytosis, likely due to multiple interactions with ACE2.^18^^,^^19^ However, in primary tissues, the levels of cathepsins are too low, and this pathway does not seem to be relevant.

Cathepsins are proteases with both endopeptidase and exopeptidase activities, involved in protein degradation in the late endosomes and lysosomes. They are classified into three catalytic types: aspartic (D and E), serine (G), and cysteine (B, C, K, L, S, and V) proteases. Among these, cysteine proteases (cathepsins B, L, and S) play the most significant role in viral entry.^20^^,^^21^^,^^22^ Cathepsin B is crucial for Ebola virus entry; cathepsin L is more relevant during coronavirus entry.^23^^,^^24^ The lower dependence of SARS-CoV-2 on the endosomal pathway explains the lack of effectiveness of endosomal acidification inhibitors, such as hydroxychloroquine, in SARS-CoV-2 therapy.^18^^,^^25^

While the use of proteases during coronaviral infection seems well-described, the analysis of the SARS-CoV-2 entry showed the unnoticed evolution of this process over time.^26^ Here, we aimed to understand this evolutionary change, and we have discovered that the preference for certain proteases changes with time due to the alteration of the protease cleavage site on the spike protein. We believe that, alongside mutations in the spike RBD, the evolution of protease preference exemplifies a continuous process that is crucial for shaping virus specificity and key characteristics such as infectivity, transmissibility, and immune evasion.

## Results

### Serine proteases are the major activating factor in the human airway epithelium

**Figure 1:**
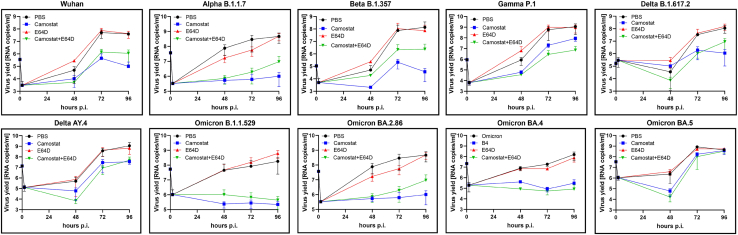
SARS-CoV-2 replication in HAEs in the presence of entry inhibitors HAE cultures were inoculated with SARS-CoV-2 (at 5000 TCID_50_ per mL) for 2 h in the presence of 100 μM camostat, 10 μM E64D, or control PBS. To analyze viral replication kinetics, each day post-infection (p.i.), 100 μL of PBS containing a given inhibitor was applied to the apical surface of the HAE cultures and collected after 10 min of incubation at 37°C. Replication of SARS-CoV-2 was evaluated by RT-qPCR analysis, and the data are presented as RNA copy numbers/ml (mean ± SD). n = 3 biological replicates.

SARS-CoV-2 S protein priming by TMPRSS2 is essential for viral entry in the natural microenvironment of the infection,^27^^,^^28^ as shown in previous studies for Wuhan, Delta, and some Omicron variants (BA.1 and XBB1.5).^29^ However, a visible change in protease requirements was observed for the omicron variant.^30^ Here, we asked whether the observed change was unique or if it was a continuous evolutionary process. For this, we analyzed virus replication kinetics in fully differentiated human airway epithelium cultures (HAEs) in the presence of TTSPs inhibitor camostat (100 μM) or cysteine cathepsins inhibitor E64D (10 μM). The results showed that all variants’ replication was efficiently blocked by camostat and not E64D; no additive or synergistic effect was recorded when a mixture of camostat and E64D was present (Figure 1). This shows that while cathepsin-mediated entry was reported on cell lines for some variants, in the primary human epithelium, TTSPs are the major activating factor.

### Protease dependence varies among SARS-CoV-2 isolates in *in vitro* models

**Figure 2:**
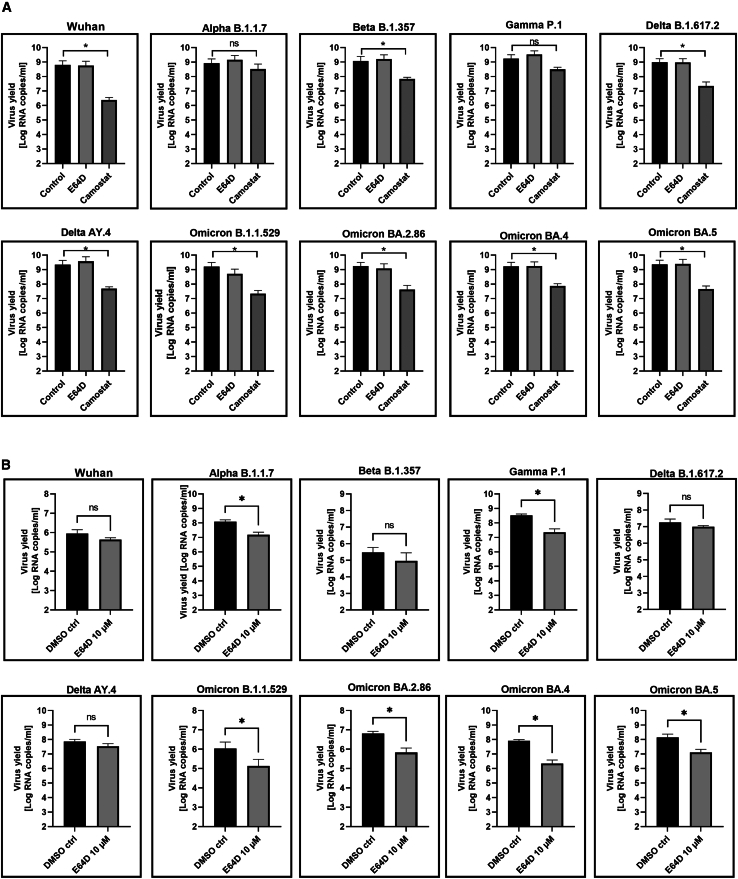
SARS-CoV-2 replication in the presence of entry inhibitors A549 cells expressing both ACE2 and TMPRSS2 (A) or A549 cells expressing only ACE2 (B) were infected with a given isolate (at 800 TCID_50_ per mL) for 2 h in the presence of 100 μM camostat (A), 10 μM E64D, or control PBS (A and B). At 96 h post inoculation, replication of SARS-CoV-2 was evaluated by RT-qPCR analysis, and the data are presented as RNA copy numbers/ml (mean ± SD). The *p* values were calculated by two-way ANOVA. (∗*p* < 0.05; ns = not significant). n = 6 biological replicates.

Knowing that *ex vivo*, all SARS-CoV-2 variants demonstrate a preference for TTSPs-mediated fusion at the cell membrane, we aimed to compare how entry inhibitors would affect the virus replication in the *in vitro* model of permissive cell line. For this, we analyzed replication of all previously used SARS-CoV-2 variants (original Wuhan, Alpha, Beta, Gamma, Delta, and Omicron) on A549 cells overexpressing ACE2 and TMPRSS2 (A549^ACE2/TMPRSS2^) in the presence of camostat (100 μM) or E64D (10 μM). In line with previous reports, original Wuhan and Delta variants were significantly inhibited by camostat, together with other isolates, including Beta and all Omicron variants, but not Alpha and Gamma. Surprisingly, we observed that the cathepsin inhibitor only weakly reduced Omicron BA.1 (B.1.1.529) replication and was ineffective in hampering infection with other isolates in these cells, showing that also in this model, TTSPs are preferred, if available (Figure 2A).

To ensure that the cathepsin-independent replication results from the TMPRSS2 expression in A549^ACE2^ cells, we repeated the experiment using cells overexpressing only ACE2 (A549^ACE2^). This time, we analyzed each variant replication in the presence of E64D or control PBS, and the results are presented in Figure 2B. In this experimental setting, we observed that Alpha, Gamma, and all Omicron isolates were significantly hampered by E64D, suggesting that virus entry was a result of a cathepsin-dependent endocytosis. Importantly, here replication of Wuhan, Beta, and Delta isolates was not affected in the absence of cathepsin activity. Furthermore, we noticed a remarkable difference in virus replication between the variants in these cells. Infection with Wuhan, Beta, and Omicron BA.1 and BA.2 was 1.5–2 log lower than Alpha, Gamma, Delta, and Omicron BA.4 and BA.5. This implied that some variants could effectively infect and replicate in A549 cells lacking activating TMPRSS2, and this process was cathepsin independent.

The results show the dependence of all SARS-CoV-2 variants on TTSPs in HAEs and, at the same time, striking differences in cathepsin and TMPRSS2 dependence; we asked a question about which other serine proteases may play a role in this process. Available literature data enabled us to select four other TTSPs of interest: human matriptase (membrane-type serine protease-1; MT-SP1), prostasin (PRSS8), hepsin (TMPRSS1), and kallikrein 13 (KLK13).^31^^,^^32^^,^^33^ A study by Beaulieu et al.^34^ showed that matriptase is involved in multicycle replication of influenza virus in human bronchial epithelial cells.^34^ Prostasin was chosen as one of the major serine proteases in human bronchial epithelial cells. KLK13 was demonstrated by us to prime HCoV-HKU1 spike for entry into HAEs. Also, other studies implicated KLKs in viral infections, such as influenza or papillomavirus.^15^^,^^35^ Lastly, hepsin (TMPRSS1) was chosen as the closest TMPRSS2-relative, belonging to the Hepsin/TMPRSS subfamily.^36^ Hepsin is predominantly expressed at highest levels in the liver, but high levels are found also in the kidneys and pancreas, what correspond to other major tissues affected by SARS-CoV-2.^37^^,^^38^^,^^39^^,^^40^

**Figure 3:**
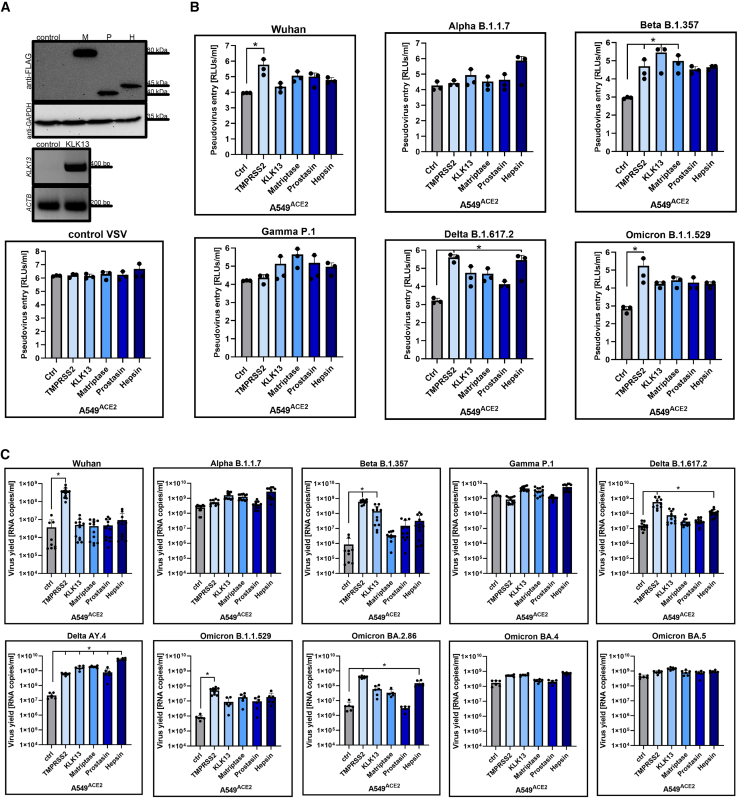
SARS-CoV-2 entry into A549 cells overexpressing ACE2 and different TTSPs (A) Lentiviral vectors were used to introduce matriptase (M), prostasin (P), hepsin (H) and Kallikrein 13 (KLK13) into A549^ACE2^ cells. (A) After antibiotic selection, cells were lysed and proteins were resolved by SDS-PAGE and analyzed by Western blotting. Each protease was detected in A549^ACE2^ cell lysates (50 μg of protein per lane) using anti-FLAG antibody (B) KLK13 mRNA was evaluated using PCR in positively transduced cells. Β-actin (ACTB) mRNA was used as an internal control. (B) Control A549 cells with ACE2 (Ctrl), A549 cells overexpressing both ACE2 and TMPRSS2 (TMPRSS2), ACE2 and KLK13 (KLK13), ACE2 and matriptase (Matriptase), ACE2 and prostasin (Prostasin), and ACE2 and hepsin (Hepsin) were transduced with HIV pseudoviruses decorated with VSV-G protein (VSV-G) or each SARS-CoV-2 variant S glycoprotein. After 72 h at 37°C, pseudovirus entry was measured by measurement of the luminescence signal in the cell lysates (relative light units [RLUs]/mL of lysate sample). The assay was performed twice, each time in triplicate (*N* = 3), and average values with standard deviation are presented. (C) Control A549 cells with ACE2 (Ctrl), A549 cells overexpressing both ACE2 and TMPRSS2 (TMPRSS2), ACE2 and KLK13 (KLK13), ACE2 and matriptase (Matriptase), ACE2 and prostasin (Prostasin), and ACE2 and hepsin (Hepsin) were infected with a given isolate (at 800 TCID_50_ per mL) for 2 h. At 96 h post inoculation, replication of SARS-CoV-2 was evaluated by RT-qPCR analysis, and the data are presented as RNA copy numbers/ml (mean ± SD). The *p* values were calculated by two-way ANOVA. (∗*p* < 0.05). n = 6 biological replicates.

Plasmids encoding each protease were introduced using lentiviral vectors into A549^ACE2^-expressing cells, which were further cultured and analyzed for protein expression by western blotting (Figure 3A). A549 with ACE2 and KLK13 overexpression (A549^ACE2/KLK13^) were prepared as shown previously.^41^ Due to the lack of kallikrein-specific antibodies, KLK13 mRNA was verified using PCR (Figure 3A). We then investigated the entry of different SARS-CoV-2 variants in the presence of various TTSPs. For this experiment, A549 cell lines were inoculated with HIV particles pseudotyped with specific SARS-CoV-2 S glycoproteins: S-Wuhan, S-Alpha, S-Beta, S-Gamma, S-Delta (B.1.617.2), S-Omicron (B.1.1.529), or a control vesicular stomatitis virus G protein (VSV-G). After three days of culture at 37°C, pseudovirus entry was quantified by measuring luciferase activity. The analysis revealed that TTSP usage is specific to each SARS-CoV-2 variant. The entry of the original Wuhan strain and the Omicron BA.1 variant was enhanced only in cells expressing TMPRSS2, whereas the Delta variant primarily entered cells with TMPRSS2 and hepsin overexpression. The Alpha variant entered only in hepsin-expressing cells. Interestingly, the Beta variant entered cells with KLK13 overexpression, and the Gamma variant showed increased entry across all cell types. Control VSV-decorated pseudoviruses entered all cell lines at the same level (Figure 3B).

Next, we aimed to analyze whether these results correspond with the entry of infectious viruses. For this, we tested replication of each SARS-CoV-2 variant in previously used A549 cell lines overexpressing certain proteases. Results are shown in Figure 3C. Notably, only the replication of the original Wuhan strain was significantly enhanced by the expression of TMPRSS2. Although the Omicron B.1 variant also exhibited this pattern, the viral titer was much lower compared to that of the Wuhan strain. The replication of the Beta variant was primarily influenced by the presence of both TMPRSS2 and KLK13. The Delta B.1 variant, and surprisingly, the Omicron BA.2 variant, showed a preference for TMPRSS2 and hepsin, while the replication of the Delta AY.4 variant was enhanced in the presence of all TTSPs. The Alpha, Gamma, and the later Omicron BA.4 and BA.5 variants replicated well across all cell lines, including control cells expressing only ACE2. This suggests that mutations in the Spike protein not only affect ACE2 recognition but may also influence the protein’s availability for activation by TTSPs.

### Molecular docking of the SARS-CoV-2 spike protein reveals differences in protein recognition and interaction

**Figure 4:**
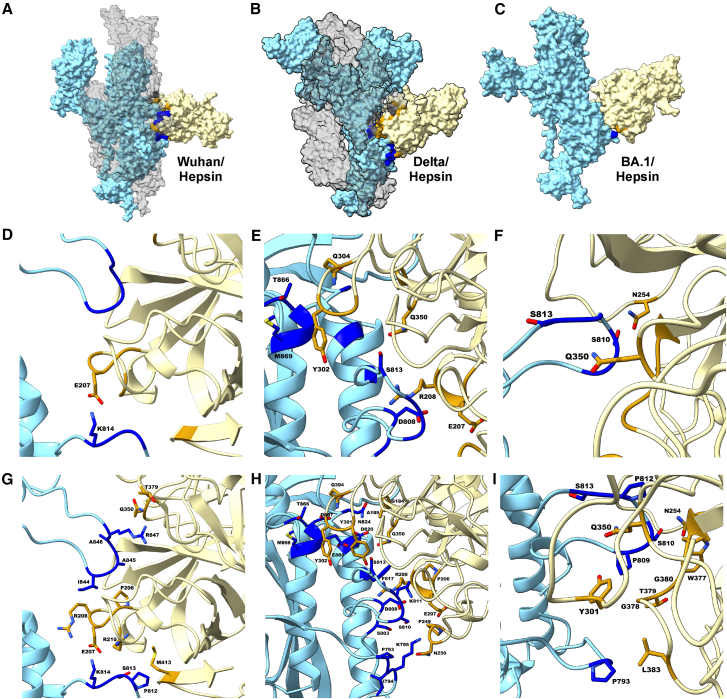
Structure and interacting residues of the Hepsin-SARS-CoV-2 spike protein complex (A–C) Surface representation of docking models showing hepsin (light yellow) bound to the spike protein from the Delta, Wuhan, and BA.1 variants. Spike protein chains are colored light blue for the main interacting chain and gray for the secondary chain (in Delta and Wuhan). Predicted interaction regions are marked in dark blue, dark gray, and red. (D–F) Close-up views of predicted stabilizing interactions, including ionic, polar, and hydrophobic contacts at the interface between hepsin and the spike protein. (G–I) Close-up views of predicted non-specific interactions, which may represent weaker or auxiliary interactions contributing to interface binding.

**Figure 5:**
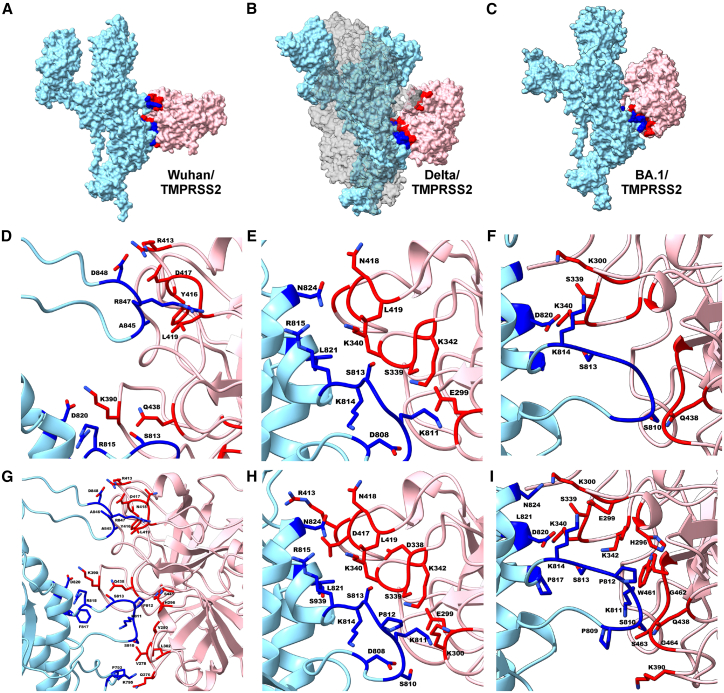
Structure and interacting residues of the TMPRSS2-SARS-CoV-2 S protein complex (A–C) Surface representation of the docking models showing TMPRSS2 (pink) bound to the spike protein from the Delta, Wuhan, and BA.1 variants. Spike protein chains are shown in light blue for the main interacting chain and in gray for the secondary chain (present only in the Delta variant). Predicted interaction regions are marked in dark blue, dark gray, and red. (D–F) Close-up view of predicted stabilizing interactions, including ionic, polar, and hydrophobic contacts between TMPRSS2 and the primary spike protein chain. (G–I) Close-up views of predicted non-specific interactions, which may reflect weaker or auxiliary interactions at the modeled interface.

This observation prompted us to conduct an *in silico* analysis to understand how different spike proteins are recognized and interact with activating proteases. We performed molecular docking of the SARS-CoV-2 S protein with two proteases, hepsin and TMPRSS2, across the Wuhan, Delta, and Omicron BA.1 variant. For each complex, we selected the top-scoring docking pose to examine the predicted protein-protein interface through contact map analysis. Figures 4A–4C and 5A–5C show these docking models for hepsin and TMPRSS2, respectively. All complexes displayed direct interactions near the S2′ cleavage site, although the involvement of one or two S protein chains varied by variant and protease. For instance, in the Wuhan/Hepsin complex, two spike protein chains contributed to the interface (Figure 4A), whereas TMPRSS2 interacted with a single chain in the Wuhan model (Figure 5A). The Delta variant showed molecular promiscuity with both proteases (Figures 4B and 5B), while the BA.1 complex involved only one spike protein (Figures 4C and 5C).

To compare the potential interface stabilization, we analyzed the residue-residue contacts that contribute the most to the protein interface stability. Table 1 reports these putative stabilizing interactions, categorized as ionic, polar, or hydrophobic. The TMPRS2-bound complexes were relatively consistent, with seven contacts for both Wuhan and Delta, and four for BA.1. For hepsin, interaction patterns differed more markedly: the Delta complex included six stabilizing contacts, whereas Wuhan and BA.1 each displayed only one or two.

**Table 1:** Specific interface contact pairs for S variants in complex with two proteases, i.e., hepsin and TMPRSS2

	TMPRSS2	Hepsin
Wuhan	**Ionic** R815-K390 D820-390K R847-D417 D848-R413	**Ionic** K814-E207
	**Polar** S813-Q438	
	**Hydrophobic** A845-Y416 A845-L419	
Delta	**Ionic** D808-K342 K811-E299 K814-K340 R815-K340	**Ionic** D808-E207 D808-R208
	**Polar** S813-S339 N824-N418	**Polar** S813-Q350 T866-Q304
	**Hydrophobic** L821-L419	**Hydrophobic** M869-Y302
BA.1	**Ionic** K814-K300 D820-K340	
	**Polar** S810-Q438 S813-S339	**Polar** S810-N254 S813-Q350

Specific contacts were determined by contact map analysis as in work by Ray et al.^42^ The main stabilizing interactions corresponding to ionic, polar, and hydrophobic contacts are presented.

The Wuhan/Hepsin complex exhibited relatively poor ionic stabilization in the docking model, with only a single ionic interaction detected, suggesting a limited electrostatic contribution to the predicted interface. This suggests a more transient or weakly stabilized interaction, potentially relying primarily on non-specific contacts. In contrast, the Delta/Hepsin complex displayed a more extensive set of stabilizing contacts, including two salt bridges, two polar contacts, and one hydrophobic interaction (Figures 4D and 4E). The combination of these interactions, particularly the salt bridges, could indicate a more favorable structural configuration at the interface. For the BA.1/Hepsin complex, stabilizing contacts near the S2*'* site were notably limited, lacking ionic and hydrophobic contributions; only two polar contacts were observed (Figure 4F).

Both the Wuhan/TMPRSS2 and Delta/TMPRSS2 complexes had four salt bridges each, suggesting a strong ionic contribution to interface stabilization. The presence of additional polar interactions within these complexes points to a more directional and likely specific binding mode with TMPRSS2, which may influence binding affinity or recognition (Figures 5D and 5E; Table 1). In contrast, the BA.1/TMPRSS2 complex exhibited fewer stabilizing features, with only two salt bridges contributing to the interaction (Figure 5F). This reduced number of stabilizing contacts may reflect the interaction efficiency and susceptibility to proteolytic activation by TMPRSS2 for this variant.

Figures 4G, 4H, 5G, and 5H, along with Figure S1, illustrate the network of predicted non-specific interactions, which generally correspond to moderate or weak hydrogen bonds occurring alongside more stabilizing contacts. In Figure S1, interactions involving residues at the S2′ site are highlighted with a black triangle. Wuhan, Delta and BA.1 variants in complex with TMPRSS2 displayed a comparable total number of predicted contacts (i.e., 12, 13, and 11, respectively). However, in the Wuhan/TMPRSS2 and BA.1/TMPRSS2 models, these interactions were restricted to a single S chain, whereas in the Delta complex, additional interactions extended to a second S chain. These additional contacts are highlighted with a star. This pattern suggests that TMPRSS2 may primarily engage with one S protein chain at the time during cleavage in the case of Wuhan and BA.1 variants, while in the Delta model, the interface may involve both chains due to their spatial proximity. A similar trend was observed in the Delta/Hepsin complex, which exhibited 25 non-specific contacts spanning both S chains, a feature less evident in the Wuhan and BA.1 complexes.

Contact map analysis and docking studies revealed distinct interaction patterns among SARS-CoV-2 variants. The Delta variant displayed a more extensive contact network near the S2*'* cleavage site compared to the Wuhan and BA.1 variant in complexes with both hepsin and, to a slightly lesser extent, TMPRSS2. These differences in contact distribution and the involvement of one or two S protein chains may reflect variant-specific binding configurations. While these *in silico* models do not directly assess cleavage or activation, they provide structural hypotheses that complement our experimental data and may help guide future mechanistic studies.

## Discussion

Evidence shows that respiratory viruses hijack host proteases to enhance and regulate cell-to-cell spread. Interestingly, such regulation improves the efficiency of infection but also restricts virus entry to the cells, which lack the expression of certain proteases. Consequently, one may state that host proteases are, yet another entry factor that pre-determines the cell, tissue, and species specificity of the virus.

Depending on the accessibility of cellular proteases needed for SARS-S activation, SARS-CoV-2 entry is mediated in two distinct ways. First, using endocytosis and activating the S protein by cathepsin L in endosomes, and second—by TTSPs if it is present with the entry receptor on the target cells’ surface.^6^ It is widely accepted that the SARS-CoV-2 Omicron variant (BA.1) altered its preference for TMPRSS2 and uses cysteine cathepsins to enter cell lines, such as Vero or Vero E6.^43^ The less efficient cleavage of S1/S2 site by furin in virus-producing cells is believed to be the major determinant of poor TMPRSS2 utilization by Omicron.^26^ However, we and others clearly show that in the biologically relevant models of human airway epithelium (HAEs), fusion of Omicron BA.1 S protein is mediated by type II transmembrane serine proteases (TTSPs). The virus entry in HAEs or intestinal organoids is easily hampered by a broad range of inhibitors of serine proteases, such as camostat, while cathepsin inhibitors do not affect virus internalization and replication.^29^ Moreover, our analysis showed that other variants, i.e., Wuhan, Alpha, Gamma, and Delta, as well as Omicron BA.2, BA.4, and BA.5 also enter HAEs via TTSPs-mediated fusion at the plasma membrane. Interestingly, identical analysis on the A549 cell line expressing ACE2 in the absence of TMPRSS2 showed a different pattern—some SARS-CoV-2 variants, such as Alpha, Gamma, and all tested Omicron isolates (BA.1, BA.3, BA.4, and BA.5) proved to be affected by E64D. Also, the replication of some of these variants in cells expressing ACE2, but not TMPRSS2, was very effective, suggesting cathepsin-dependent endocytosis being the primary entry route. Interestingly, in A549^ACE2/TMPRSS2^ cells, cathepsin inhibitors did not have such a strong effect, as only Omicron BA.1 replication slightly declined. Apparently, in A549 cells, in the absence of TMPRSS2, some SARS-CoV-2 variants carry the ability to hijack the endocytic entry pathway. This effect is not observed in *ex vivo* tissues, presumably due to lower cathepsin expression levels. However, a recent study by Kakizaki M. et al.^44^ demonstrated that while TMPRSS2 is essential for SARS-CoV-2 infection in certain cells and animal models, other proteases, such as cysteine cathepsins and serine proteases, also play critical roles in facilitating infection within human respiratory organoids.^44^ These results highlight that SARS-CoV-2 employs a more intricate mechanism involving multiple proteases to infect human airways. This mechanism appears to be more sophisticated than those observed in traditional cell lines or animal models, potentially explaining how different variants achieve effective transmission and infection in humans.

Many reports demonstrate that the SARS-CoV-2 Omicron variant shows less efficient replication and fusion activity than the Delta variant in TMPRSS2-expressed cells.^45^ This aligns with our observations, where the Omicron BA.1 titer obtained in A549^ACE2/TMPRSS2^ cells was ∼1–1.5 log lower than Wuhan. A study by Benlarbi et al.^46^ showed that in the absence of TMPRSS2, SARS-CoV-2 spike proteins of Delta and Omicron can use the matrix metalloproteinases (MMPs), MMP-2 and 9, to induce cell-cell fusion. Also, in cells expressing high levels of MMP-2/9, infection and syncytia formation induced by native SARS-CoV-2 Alpha were significantly reduced by MMP inhibitors. Authors suggested that in the context of hyperinflammation and dysregulated immune responses, MMPs could play a role in facilitating SARS-CoV-2 viral entry and syncytia formation, expanding tropism to TMPRSS2 negative cells, and exacerbating COVID-19.^46^ Here, we aimed to examine the SARS-CoV-2 Spike evolution in the context of its recognition by other TTSPs.

Transmembrane serine proteases occupy a vital role in numerous physiological processes.^47^ These enzymes are characterized by an extracellular C-terminal domain with serine protease activity, a single transmembrane domain, and a short cytoplasmic N-terminal domain. The human TTSP family consists of 17 members, categorized as a few subfamilies. The HAT/DESC subfamily includes HAT, DESC1, TMPRSS11A, and several HAT-like proteins, all having a SEA domain in their stem region. Hepsin/TMPRSS subfamily, which comprises hepsin, TMPRSS2, TMPRSS3, TMPRSS4, TMPRSS5/spinesin, MSPL, and enteropeptidase. Lastly, the matriptase subfamily contains Matriptase, Matriptase-2, Matriptase-3, and Polyserase-1.^10^^,^^36^ Kallikreins (KLKs) represent another interesting family of TTSPs; the recent data suggest that extracellular proteolysis mediated by KLKs is an important regulator of certain viral infections KLK5 and KLK12 were shown to cleave and expose the fusogenic fragment of hemagglutinin (HA) protein in the influenza viral envelope to render viral particles infectious. Also, in our study, KLK13 was proved to be responsible for the activation of the HCoV-HKU1 S protein, both in cell culture and in the natural environment of the respiratory tract.^41^ A recent study by Kim H. et al.^48^ showed the role of kallikreins in activating beta-coronaviruses, including SARS-CoV-2.^48^

In our study, we expressed some of these proteases on A549 cells expressing the ACE2 receptor protein and analyzed SARS-CoV-2 entry and replication in the presence of different TTSPs. We found that SARS-CoV-2 variants exhibit different preferences toward specific proteases. The original Wuhan strain and Omicron BA.1 used only TMPRSS2 for entry and replication, while Beta or Delta isolates preferred KLK13 or hepsin, respectively. To prove that the observed effect is not affected by protein overexpression, we carried out *in silico* studies on the S protein interaction with the host cell proteases via molecular docking and contact map analysis. Our results clearly show that the Delta variant exhibits a more extensive contact network of interaction at the S2*'* cleavage site with both hepsin and TMPRSS2 compared to the Wuhan and BA.1 variants. We noted that different SARS-CoV-2 variants vary in their binding stability with those proteases, impacting S protein activation and, thus, cell entry efficiency. The Delta variant shows notably stronger and more efficient binding to hepsin due to additional salt bridges, polar contacts, and hydrophobic interactions, enhancing stability and specificity. In contrast, the BA.1 variant’s interaction with hepsin lacks ionic and hydrophobic stabilization, relying solely on two polar contacts, leading to a weaker, potentially less stable binding. For interactions with TMPRSS2, both the Wuhan and Delta variants display strong ionic stabilization, each forming four salt bridges along with additional polar contacts, suggesting a stable and specific binding interface that may enhance proteolytic activation. The BA.1 variant, however, shows reduced stabilization with TMPRSS2, exhibiting only two ionic interactions, which could lower interaction efficiency and impact proteolytic susceptibility. This clearly showed that the Delta S protein S2-S2*'* site is better recognized by Hepsin, than TMPRSS2, in contrast to Wuhan S2-S2′ region.

Mutations in the SARS-CoV-2 S protein are central to the virus’s evolution, as the spike plays a critical role in mediating viral entry into host cells and determining infectivity, transmissibility, and immune evasion. Altered characteristics of a given isolate may result from mutations within RBD, as well as the evolution of the S cleavage site, determining protease usage for cell entry. While furin cleavage site mutations can enhance the virus’s ability to infect human cells, different SARS-CoV-2 variants exhibit changes that allow them to shift between TMPRSS2 and other cellular proteases, adapting to different tissue types. This was most clearly observed for Omicron BA.1, in which infectivity was significantly increased in human nasal epithelial cells, a primary entry point for SARS-CoV-2.^49^ The combination of increased infectivity in nasal tissues and immune evasion mechanisms likely explains Omicron’s rapid global spread and dominance over earlier variants.

To summarize, we believe that the evolution of the S-SARS-CoV-2 cleavage site is an important and previously overlooked factor modifying the disease severity and transmissibility. The emergence of omicron yielded a milder yet more transmissible virus, which in turn determined its evolutionary success and, together with growing immunity in the society, terminated the severe phase of the pandemic.^50^^,^^51^^,^^52^ Understanding the genotype-phenotype relationship is pivotal for anticipating the clinical and epidemiological impacts of newly emerging virus variants. Given the widespread requirement of protease-mediated entry by diverse viruses, including the influenza virus, we propose that it is essential to include such analyses to link the genotype and phenotype. We propose that such an approach may enhance our ability to predict changes in infectivity, transmissibility, and pathogenicity of emerging variants and viruses.

### Limitations of the study

This study was designed to investigate the molecular flexibility of the SARS-CoV-2 spike protein in terms of its activation by host proteases beyond the commonly studied TMPRSS2. The selection of alternative proteases, i.e., matriptase, Kallikrein 13, prostasin, and hepsin was based on their expression in human airway epithelial cells. However, it must be noted that the human serine protease repertoire is far more extensive, with around 200 serine proteases potentially expressed in the respiratory tract. Therefore, while our analysis highlights the potential role of additional proteases, it does not capture the full spectrum of host enzymes that may contribute to spike activation *in vivo*. Hepsin was included in our *in vitro* assays due to its airway expression, and our results suggest that some viral strains may be more finely tuned for activation by hepsin or other proteases rather than TMPRSS2, owing to mutations that affect both the amino acid sequence and the conformational dynamics of the spike protein. Nevertheless, based on these *in vitro* experiments alone, we cannot definitively conclude that hepsin plays a significant role *in vivo*, and further studies will be needed to validate its relevance under physiological conditions.

## Resource availability

### Lead contact

Further information and requests for resources and reagents should be directed to and will be fulfilled by the lead contact, Krzysztof Pyrc (k.a.pyrc@uj.edu.pl).

### Materials availability

This study did not generate new unique reagents.

### Data and code availability

All data reported in this paper will be shared by the lead contact upon request. Atom simulations snapshots and contact maps analysis scripts for this work are available at https://doi.org/10.5281/zenodo.3817446 and also at https://doi.org/10.5281/zenodo.14033250. Any additional information required to reanalyze the data reported in this paper is available from the lead contact upon request.

## Acknowledgments

This study was generated in the context of the DURABLE project, co-funded by the European Union, under the EU4Health Program (EU4H), project no. 101102733 to KP; Opus Grant (UMO-2017/27/B/NZ6/02488 to K.P.); and ERAnet ICRAD—project Musecov: Multi-scale Eco-evolution of Coronaviruses: from surveillance toward emergence prediction (to K.P.). The open-access publication of this article was funded by the Priority Research Area BioS under the program “Initiative of Excellence – Research University” at the Jagiellonian University in Krakow.

Views and opinions expressed are however those of the authors only and do not necessarily reflect those of the European Union or the European Health and Digital Executive Agency. Neither the European Union nor the granting authority can be held responsible for them. A.B.P. acknowledges financial support from the National Science Center, Poland, under grant 2022/45/B/NZ1/02519 and gratefully acknowledges Polish high-performance computing infrastructure PLGrid (HPC Centers: ACK Cyfronet AGH) for providing computer facilities and support within computational grant no. PLG/2025/018510.

The authors sincerely thank Prof. Johan Neyts (Virology at the University of Leuven, KU Leuven, Belgium), Prof. Etienne Simone-Loriere (Institute Pasteur, Paris, France), and Dr. Helena Jiřincová (National Institute of Public Health, Prague, Czech Republic) for sharing SARS-CoV-2 isolates.

## Author contributions

A.M., experimental design, carrying out experiments, data analysis, figure preparation, and manuscript writing. L.F.C.-V., carrying out experiments, data analysis, and figure preparation. A.B.P. and K.P., experimental design, obtaining funding, and manuscript writing.

## Declaration of interests

The authors declare no conflict of interest.

## STAR★Methods

### Key resources table

**Table:** 

REAGENT or RESOURCE	SOURCE	IDENTIFIER
**Antibodies**		

Monoclonal ANTI-FLAG® M2 antibody produced in mouse	Sigma-Aldrich	Cat# F3165; RRID: AB_259529
Polyclonal Rabbit Anti-Mouse Immunoglobulins/HRP	Dako	Cat#P0260

**Bacterial and virus strains**		

SARS-CoV-2 Wuhan	EVAg	Munchen-1.2 2020/984
SARS-CoV-2 Alpha B1.1.7	EVAg	hcov19/sweden/20-53840/2020
SARS-CoV-2 Beta B1.351	EVAg	hcov19/sweden/21-53217/2021
SARS-CoV-2 Gamma P.1	EVAg	20J/501Y.V3
SARS-CoV-2 Delta B.1.617.2	EVAg	7102/21 Kmen
SARS-CoV-2 Delta AY.4	a gift from Helena Jiřincová	9377/21 Kmen
SARS-CoV-2 Omicron B.1.1.529	a gift from Helena Jiřincová	England/M02-22/2022
SARS-CoV-2 Omicron BA.2.86	a gift from Etienne Simone-Loriere	hu/DK/SSI-H135
SARS-CoV-2 Omicron BA.4	a gift from Johan Neyts (KU Leuven)	1-JE22
SARS-CoV-2 Omicron BA.5	a gift from Johan Neyts (KU Leuven)	1-GPE-27

**Biological samples**		

Human Airway Epithelial Cells, Bronchial origin	Epithelix Sarl	EP51AB

**Deposited data**		

SARS-CoV-2-Wuhan Spike 3D coordinates	National Institute of Allergy and Infectious Diseases Data Ecosystem	https://data.niaid.nih.gov/resources?id=zenodo_3817446

**Experimental models: Cell lines**		

A549	ATCC	CCL-185
HEK 293T	ATCC	CRL-3216
Vero	ATCC	CCL-81

**Oligonucleotides**		

SARS-CoV-2 RT-qPCR 5′ primer	Genomed, Poland	CACATTGGCACCCGCAATC
SARS-CoV-2 RT-qPCR 3′ primer	Genomed, Poland	GAGGAACGAGAAGAGGCTTG
SARS-CoV-2 RT-qPCR fluorescent probe	Genomed, Poland	6-ACTTCCTCAAGGAAC AACATTGCCA-BHQ-1

**Recombinant DNA**		

pCMV6-Matriptase	OriGene Technologies	Cat#RC207136
pCMV6-Prostasin	OriGene Technologies	Cat#RC200646
pCMV6-Hepsin	OriGene Technologies	Cat#RC221292
psPAX2	Addgene	Cat#12260
pMD2.G	Addgene	Cat#12259
pRRL Luciferase	Addgene	Cat#120798
SARS-CoV-2-S-Wuhan	GeneArt	custom vector
SARS-CoV-2-S-Alpha	Addgene	Cat#170451
SARS-CoV-2-S-Beta	Addgene	Cat#170449
SARS-CoV-2-S-Gamma	Addgene	Cat#170450
SARS-CoV-2-S-Delta	Addgene	Cat#155130
SARS-CoV-2-S-Omicron	Addgene	Cat#180375

**Software and algorithms**		

HADDOCK v2.5-2024.03 webserver	HADDOCK 2.4 https://rascar.science.uu.nl/haddock2.4/	HADDOCK v2.5-2024.03
Modeller v10.5	Sali et al.^53^	Modeller v10.5
AMBER22	Case et al.^54^	AMBER22
UCSF ChimeraX v1.8	Meng et al.^55^	UCSF ChimeraX v1.8
Contact map analysis	Ray et al.^42^	OV+rCSU

### Experimental model and study participant details

#### Plasmid constructs

Plasmids encoding FLAG-tagged Matriptase, Prostasin and Hepsin were purchased in Origene™ Technologies. Inserts encoding each protease were subcloned into the pWPI plasmid for lentivirus production, and sequences were verified with sequencing.

#### Cell culture

##### Cell lines

A549 (ATCC CCL-185), A549 cells overexpressing ACE2 (A549^ACE2^), A549 cells overexpressing both ACE2 and TMPRSS2 (A549^ACE2/TMPRSS2^), Vero cells (ATCC: CCL-81) and HEK293T cells (ATCC CRL-3216) were maintained in Dulbecco’s modified Eagle’s medium (DMEM, high glucose; Thermo Fisher Scientific, Warsaw, Poland) supplemented with 5% heat-inactivated fetal bovine serum (5% DMEM; Thermo Fisher Scientific), penicillin (100 U/mL; Thermo Fisher Scientific), and streptomycin (100 μg/mL; Thermo Fisher Scientific). A549^ACE2/TMPRSS2^ cells were supplemented with blasticidin S (10 μg/mL, Sigma-Aldrich), and puromycin (0.5 μg/mL, Sigma-Aldrich) to maintain the ACE2+TMPRSS2+ population. Cells were cultured at 37°C, 5% CO2, and 95% humidity. All cell lines were tested every two weeks for mycoplasma contamination either by in-house DAPI staining or LookOut® Mycoplasma PCR Detection Kit (Sigma-Aldrich).

##### Human epithelium (HAE) cultures

Human airway epithelial cells (Epithelix SAS, Archamps, France) were maintained in BEGM medium. Before the test, cells were trypsinised and transferred to a permeable transwell insert supports (f = 6.5 mm). Cell differentiation was stimulated by medium additives. The removal of the media from the apical side was performed after the cells reached confluence. Cells were cultured for 3 to 5 weeks to form well-differentiated, pseudostratified mucociliary epithelia, as previously described.^41^^,^^56^

##### Virus stocks

SARS-CoV-2 isolates: Wuhan (Munchen-1.2 2020/984), Alpha (B1.1.7), Beta (B.1.357), Gamma (P.1), Delta (B.1.617.2 and AY.4) and Omicron (B.1.1.529, B.2.86, BA.4 and BA.5) were generated by infecting monolayers of Vero cells. The virus-containing liquid was collected on day 3 postinfection (p.i.), aliquoted, and stored at -80°C. Control Vero cell lysate from mock-infected cells was prepared in the same manner. Plates were incubated at 37°C for 3 days, and the cytopathic effect (CPE) was scored by observation under an inverted microscope. Virus yield was assessed by titration on fully confluent Vero cells in 96-well plates, according to the method of Reed and Muench (1938). All viral work was performed in a Biosafety Level 3+ laboratory (BSL3+).

### Method details

#### Pseudovirus production and transduction

HEK 293T cells were seeded in 10-cm diameter dishes, cultured for 24 h at 37°C with 5% CO2, and transfected using polyethylenimine (Sigma-Aldrich, Saint Louis, USA) with the lentiviral packaging plasmid (psPAX), the VSV-G envelope plasmid (pMD2G), or SARS-CoV-2 S glycoprotein (pCAGGS-SARS-CoV-2-S) and a third plasmid encoding firefly luciferase protein (pRRL luciferase). Cells were further incubated for 72 h at 37°C with 5% CO2, and pseudoviruses were collected every 24 h and stored at 4°C. A549^ACE2/TMPRSS2^ cells were seeded in 96-well plates, incubated for 24 h at 37°C with 5% CO2, and transduced with pseudoviruses harbouring VSV-G or S-SARS-CoV-2 proteins or lacking the fusion protein (ΔEnv) or in the presence of Polybrene (Sigma-Aldrich, Saint Louis, USA) (4 mg/ml). After 4 h of incubation at 37°C, the unbound virions were removed by three washes with PBS, and cells were further incubated for 72 h at 37°C with 5% CO2. After that, cells were lysed using Bright-Glo luciferase assay buffer (Promega, Poland) and transferred to white 96-well plates. Luminescence levels were measured on a SpectraMAx iD5 microplate reader (Molecular Devices, San Jose, California, USA).

#### Lentivirus production and transduction

HEK 293T cells were seeded on 10-cm2 dishes, cultured for 24 hours at 37°C with 5% CO2, and transfected with psPAX, pMD2G, and third transfer plasmid (pWPI/Matriptase, pWPI/Prostasin, pWPI/Hepsin) with polyethyleneimine (Sigma-Aldrich, Poland). Both psPAX (Addgene plasmid no. 12260) and pMD2G (Addgene plasmid no. 12259) were gifts from D. Trono). The cells were further cultured for 96 hours at 37°C with 5% CO2, and lentiviral particles were collected every 24 hours and stored at 4°C. Lentivirus stocks were concentrated 25-fold with centrifugal protein concentrators (Amicon Ultra, 10-kDa cutoff; Merck, Poland) and stored at −80°C. A549^ACE2^ cells were seeded in T75 flasks, cultured for 24 hours at 37°C with 5% CO2, and transduced with lentiviral particles expressing each protease in the presence of polybrene (4 μg/ml; Sigma-Aldrich, Poland). Cells were further cultured for 72 hours at 37°C with 5% CO2, and positively transduced cells were selected with blasticidin (2 μg/ml; Sigma-Aldrich, Poland).

#### Detection of Matriptase, Prostasin and Hepsin

After they underwent selection in blasticidin-containing medium, A549^ACE2^ cells expressing Matriptase (A549^ACE2/M^), Prostasin (A549^ACE2/P^) and Hepsin (A549^ACE2/H^) or control cells (A549^ACE2^) were scraped and collected by centrifugation. Cells were lysed in radioimmunoprecipitation assay (RIPA) buffer [50 mM tris, 150 mM NaCl, 1% Nonidet P-40, 0.5% sodium deoxycholate, and 0.1% SDS (pH 7.5)], boiled for 5 min, cooled on ice, and resolved on a 10% polyacrylamide gel alongside dual-colour Page Ruler Prestained Protein size markers (Thermo Fisher Scientific, Poland). The separated proteins were then transferred onto a Westran S polyvinylidene difluoride (PVDF) membrane (GE Healthcare, Poland) by wet blotting (Bio-Rad, Poland) for 1 hour at 100 V in transfer buffer (25 mM tris, 192 mM glycine, and 20% methanol) at 4°C. The membranes were blocked by overnight incubation at 4°C in Tris-buffered saline (TBS)–Tween (0.1%) buffer supplemented with 5% skimmed milk (BioShop, Canada). The blots were incubated with a mouse monoclonal anti–FLAG antibody (clone P5H9-A3; 1:500 dilution; Sigma-Aldrich, Poland), followed by incubation with a horseradish peroxidase (HRP)–labeled anti-mouse immunoglobulin G (65 ng/ml; Dako, Denmark) diluted in 5% skimmed milk/TBS-Tween (0.1%). The signal was developed with the Pierce ECL Western blotting substrate (Thermo Fisher Scientific, Poland) and visualized with the ChemiDoc Imaging System (Bio-Rad, Poland).

#### Virus infection

In *in vitro* experiments, fully confluent A549^ACE2^, A549^ACE2/TMPRSS2^, A549^ACE2/KLK13^, A549^ACE2/M^, A549^ACE2/P^, A549^ACE2/H^ cells in 96-well plates were exposed to either SARS-CoV-2 or a mock treatment at a 50% tissue culture infective dose (TCID_50_) of 800 per ml in the presence of tested inhibitor or control medium. After 2 h incubation at 37°C, unbound virions were removed by washing with 100 μl of PBS and fresh medium with a given compound was added to each well. Cell culture supernatants were collected on day 4 p.i. and analysed using reverse transcription-quantitative PCR (RT-qPCR). For the *ex vivo* study, fully differentiated human airway epithelium cultures were exposed to the tested inhibitor or the control (PBS) for 60 min at 37°C, following inoculation with SARS-CoV-2 at a TCID_50_ of 800 per ml in the presence of the inhibitor or control PBS. After 2 h incubation at 37°C, unbound virions were removed by washing with 200 μl of PBS, and HAE cultures were maintained at the air-liquid interface till the end of the experiment. To analyse the kinetics of the virus replication, each day p.i., 100 μl PBS with a given inhibitor or control PBS was applied on the apical surface of the HAE and collected after a 10-min incubation at 37°C. All samples were stored at -80°C and analysed using RT-qPCR.

#### Isolation of nucleic acids and reverse transcription-quantitative PCR (RT-qPCR)

Isolation of viral RNA was carried out using a commercially available RNA isolation kit (MagnifiQ™ 96 Pathogen instant kit; A&A Biotechnology, Poland) and KingFisher Flex System (Thermo Fisher Scientific, Poland), according to the protocol provided by the manufacturer. The isolated RNA was subjected to RT-qPCR using the GoTaq® Probe 1-Step RT-qPCR System Protocol kit (Promega, Madison, Wisconsin, USA) according to the manufacturer’s instructions with the use of primers and probes (Forward: 5′-CACATTGGCACCCGCAATC-3’; Reverse: 5′-GAGGAACGAGAAGAGGCTTG-3′; Probe: 5′-6-ACTTCCTCAAGGAACAACATTGCCA-BHQ-1-3′). Appropriate standards were prepared to evaluate the number of viral RNA molecules in the samples. The reaction was carried out in a thermal cycler (CFX96 Touch Real-197 Time PCR Detection System; Bio-Rad, Hercules, California, USA).

#### Protein-protein docking

Molecular docking between the SARS-CoV-2 S protein of Wuhan, Delta (PDB: 7W92),^57^ and BA.1 (PDB: 7XO5)^58^ and the human proteases TMPRSS2 (PDB: 8HD8)^59^ and Hepsin (PDB: 1Z8G)^60^ was performed using the HADDOCK v2.5-2024.03 webserver.^61^^,^^62^ Missing loops in the protein structures were reconstructed using Modeller v10.5.^53^ Every heteroatom was removed from the reference structures. The relaxed atomistic coordinates for each S protein variant were derived via all-atom molecular dynamics (MD) simulations. These simulations were performed using AMBER22 with the FF19SB force fields and the pmemd.cuda module for enhanced performance.^63^^,^^64^ For the Wuhan variant the S protein was retrieved from our previous modelling study,^65^ where for Delta and BA.1, each S protein was placed in a dodecahedral box, extending 20 Å beyond the solute in every cartesian direction, and solvated with the four-site OPC water model.^63^ The systems were neutralized with counterions, specifically one Cl− ion for the Delta variant and three Cl- ions for the BA.1 variant. To remove local clashes, a geometric optimization was performed using the steepest descent algorithm for 5000 cycles. The MD equilibration process consisted of several stages. First, temperature equilibration in the NVT ensemble was performed by gradually increasing the temperature through steps of 150, 200, 250, 300, and finally 310 K, each lasting 200 ps. During this phase, position restraints were applied to the heavy atoms of the proteins, with progressively decreasing spring constants of 5.0, 4.0, 3.0, and 1.0 kcal mol−1 Å−2, facilitating gradual relaxation. This was followed by a 1 ns equilibration at 310 K in the NPT ensemble without restraints. For production MD, the simulations were run in the NPT ensemble with periodic boundary conditions and Particle Mesh Ewald (PME) method^66^^,^^67^ a grid spacing of 1.0 Å for long-range electrostatics. Non-bonded interactions were modelled with a Lennard-Jones potential using a 9Å cutoff. Temperature control was maintained using Langevin dynamics^68^ with a collision frequency of 4.0 ps−1, and pressure control was managed by the Monte Carlo barostat^69^ with a 2.0 ps relaxation time at 1 bar. Bond constraints on hydrogen atoms were applied using the SHAKE algorithm,^70^ and the hydrogen mass repartitioning scheme was applied via ParmEd,^71^ enabling a 4fs integration time step.^72^ Each protein complex was simulated for a total of 20 ns. For the Wuhan variant, the 3D coordinates were retrieved from.^73^

The active interaction region on the spike protein was defined as the cleavage site (residues P809-R815). For TMPRSS2 and Hepsin, the active sites were defined based on their catalytic residues: H296, D345, D435, S441, S460, and G462 for TMPRSS2, and H203, D257, D347, A348, and S353 for Hepsin. These specific regions were selected to guide the docking process and maximize biologically relevant interactions. Docking clusters were analyzed by selecting those with the lowest interaction energies for further structural analysis. To evaluate binding accuracy, native contacts between the S protein and proteases were computed using the contact map analysis based on the OV+rCSU method,^42^^,^^74^ which allows for a precise identification of critical stabilizing interactions, both specific and non-specifics. High-frequency contacts, defined as those appearing in over 70% of the generated models, were highlighted as key determinants of protein-protein recognition, providing insight into the most stable and consistent interactions across docking configurations. For visualization, the selected models were rendered using UCSF ChimeraX v1.8.^55^

### Quantification and statistical analysis

Each graph is representative of at least three independent virus infection experiments, each performed with a minimum of three biological replicates. Experiments on human airway epithelial cells were performed on cells obtained from three different donors. Statistical analyses were performed using GraphPad Prism 8. The Shapiro-Wilk test was used to evaluate normality of all data. Groups were compared by one-way- or two-way-ANOVA. The details of replicate number, dispersion and precision measures for each experiment are given in the corresponding Figure legend.
