# Supplementary material for: Evolution of the SARS-CoV-2 spike protein in utilizing host transmembrane serine proteases
Source: iScience. 2025 Aug 6;28(9):113318. doi: 10.1016/j.isci.2025.113318 (PMC12496188; doi:10.1016/j.isci.2025.113318)
Supplement: Document S1. Figure S1 [file mmc1.pdf]

iScience, Volume *28*

Supplemental information

Evolution of the SARS-CoV-2 spike protein in utilizing host transmembrane serine proteases

Aleksandra Milewska, Luis Fernando Cofas-Vargas, Adolfo B. Poma, and Krzysztof Pyrć

Supplementary information

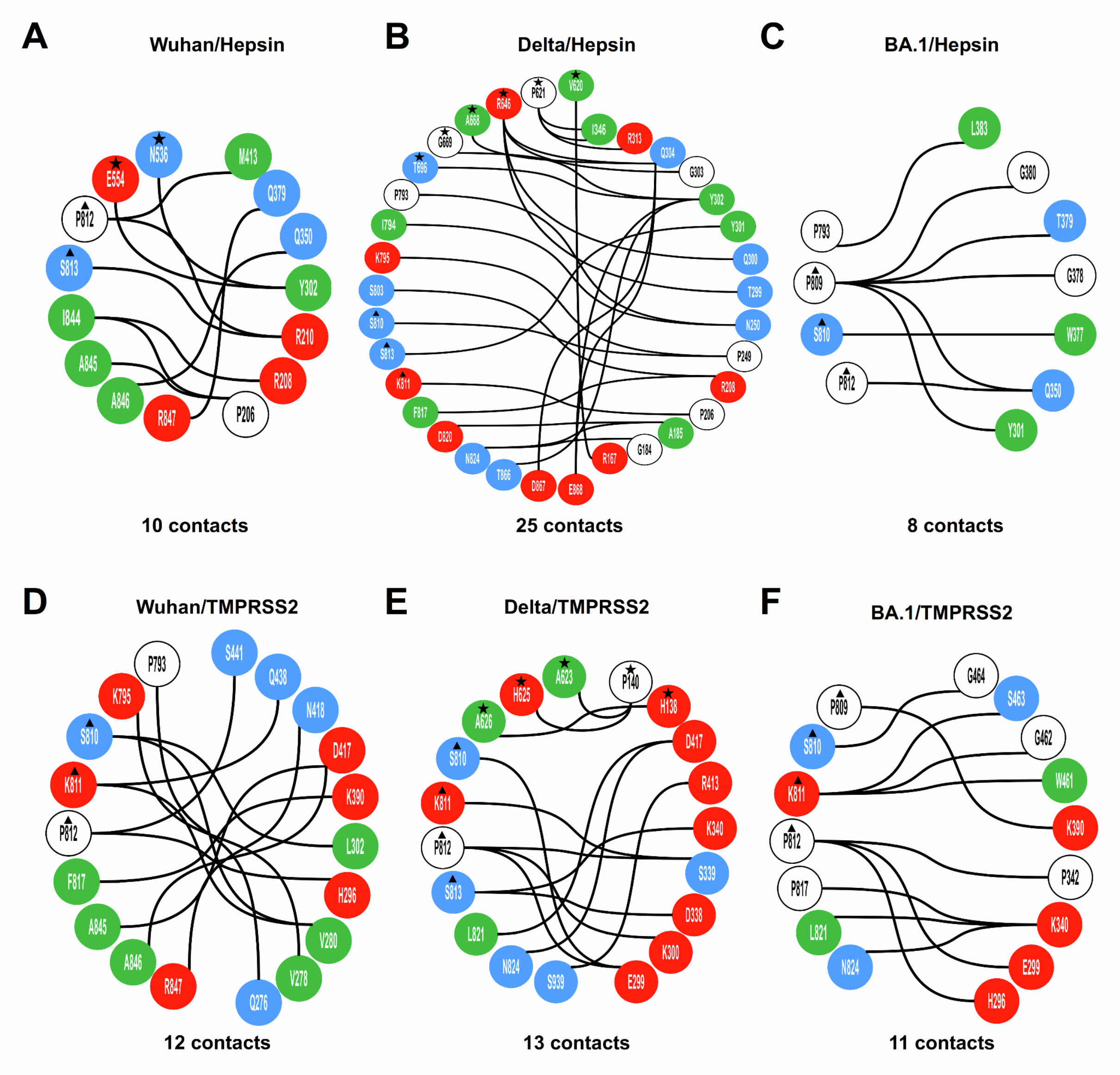

Figure S1. Network representation of the non-specific contacts for the S protein variant/protease complexes at the interface. The black lines represent pairs of contacts. The amino acid residues are coloured based on their chemical properties. The star above the residue name indicates that a given residue is found in another S protein chain. Triangles indicate residues in the S2' site.
